# Trust Game Database: Behavioral and EEG Data From Two Trust Games

**DOI:** 10.3389/fpsyg.2019.02656

**Published:** 2019-12-12

**Authors:** Chao Fu, Xiaoqiang Yao, Xue Yang, Lei Zheng, Jianbiao Li, Yiwen Wang

**Affiliations:** ^1^School of Economics and Management, Fuzhou University, Fuzhou, China; ^2^Center for China Social Trust Research, Fuzhou University, Fuzhou, China; ^3^Institute of Psychological and Cognitive Sciences, Fuzhou University, Fuzhou, China; ^4^School of Economics, Institute for Study of Brain-Like Economics, Shandong University, Jinan, China

**Keywords:** one-shot trust game, iterated trust game, electroencephalogram, event-related potentials, time-frequency power

## Background and Summary

The trust refers to the willingness of an individual to voluntarily offer his/her personal resources to others based on the positive expectation of their behaviors or intentions in an uncertain social situation and therefore put himself/herself in a weak position (Mayer et al., 1995; Rousseau et al., 1998; Giovanna et al., 2013; Thielmann and Hilbig, 2015). The trust is not only the precondition to establish and maintain cooperative relationships (Davis et al., 2000; Krueger et al., 2007) but also the footstone of the benign and orderly operation of society (Tzieropoulos, 2013). Thus, it is not surprising to witness a rapidly growing body of trust research across different disciplines of social sciences (e.g., economics, sociology, political sciences, and psychology). Although above studies have highlighted that the trust generates various economic, social, and political payoffs for modern societies (Knack and Keefer, 1997; Newton, 2001), researchers have consistently failed to reach consensus with respect to the most basic issue in trust field, that is, what motivates people to trust or distrust with unknown others?

In recent years, with the development of brain imaging technology, many researchers have tried to answer the above question from the perspective of the neural mechanism of trust. Due to the non-invasive and high time resolution characteristics (i.e., millisecond, Wang et al., 2011; Mu et al., 2016), the electroencephalogram (EEG) was widely used in such trust-related studies to examine the neural dynamics of trust behaviors (Wang et al., 2016, 2017). However, compared to behavioral research, the EEG study often takes more time and money. If these data can be reused, it will not only saves a lot of manpower and financial resources but also facilitates the comparison and validation of the results of participants with different backgrounds (Poldrack and Gorgolewski, 2014; Shin et al., 2018). Regrettably, so far, trust-related open brain imaging databases are relatively few. Thus, the aim of the current data report is to provide such an open-access database to share our data with potential researchers in this field.

Specifically, the trust game is a classic game paradigm for studying trust behaviors in laboratories. According to whether the trustee remains the same person, the trust game can be turned into a one-shot trust game (OTG) or an iterated trust game (ITG). In an OTG, the trustor's decisions are in response to a different trustee in each round, which simulated the trust behaviors among strangers. While in an ITG, each trustor plays with the same trustee over multiple rounds, and this simulated the trust behaviors among acquaintances (Rousseau et al., 1998; Buskens et al., 2016). Current Trust Game Databases provided the demographic data, behavioral data and raw EEG data (.cnt format; Neuroscan Inc.) of 40 healthy Chinese participants while they played the role of the trustor in OTG or ITG. In addition, given the decision-making stage and the outcome-feedback stage are the two most important stages during the trust decisions (Platt, 2002; Paulus, 2005). To facilitate the reuse of our database, based on the processing of raw EEG data, we further provided the ERPs data and time-frequency power data of the decision-making stage and the outcome-feedback stage, respectively. We hope that our databases will bring convenience to relevant researchers and laboratories.

## Methods

### Participants

To exclude the potential EEG differences caused by the relative hemispheric dominance or “handedness” of the individual (Murri, 1984; Nielsen et al., 1990), forty self-reported right-handed healthy Chinese undergraduate and graduate students were recruited. Of whom, 20 participants (11 females) performed the OTG and the other 20 participants (11 females) performed the ITG. All participants had a normal or corrected-to-normal vision, and none had a history of any neurological, psychiatric, or other brain-related diseases that might affect the results. All participants were informed about the experimental protocol and were financially reimbursed after the experiment. In accordance with the Helsinki Declaration of Human Rights (World Medical Association, 1975), written informed consent was obtained from all participants after a detailed explanation of the study. The research protocol was approved by the local Ethics Committee and was in compliance with the ethical standards of the American Psychological Association.

### Experimental Task

The current experimental task was modified from Berg et al.'s trust game (Berg et al., 1995) and has two versions, namely, the OTG wherein the trustor's decisions are in response to a different completely anonymous trustees in each round, and the ITG wherein the trustor plays with the same trustee over multiple rounds. In the current data report, the participants played the role of the trustor with the alleged trustee in both OTG and ITG. At the beginning of each round, both the trustor and the trustee are given 10 game points as an initial endowment. The trustor was needed to decide at first whether to send all his/her 10 points to the trustee or to keep this endowment. If the trustor chooses to keep it, this round ends and both players will receive 10 points. If the trustor chooses to send the initial endowment, this 10 points will be tripled to 30 points and sent to trustee, and the trustee will then decides how to allocate these tripled points plus his/her own initial endowment (30 + 10 = 40 points in total). The trustee has also two options: to divide the 40 points equally and send back 20 points to the trustor or to keep the 40 points and send nothing back. Given the possibility of being exploited by the trustee, the trustor's decision to send money reflects his/her willingness to be vulnerable to the trustee's allocation decision, which is the behavioral operationalization of trust.

In the OTG, participants were informed that in each round the trustee was a different adult randomly selected from a large and representative subject pool (*N* = 400), and the experimenter sampled and interviewed these adults before this experiment. Participants were told that these adults were asked to imagine participating in a single round trust game and were asked to indicate their choices between sending 20 points back and keeping all 40 points if they were entrusted 30 points by a stranger. Participants were also told that the experimenter recorded all these adults' choices and the computer would randomly select one from all these choices to respond to the participant's choice in each round. In the ITG, participants were informed that in each round, the trustee was the same adult randomly selected from a large and representative subject pool (*N* = 400), and the experimenter sampled and interviewed these adults before the experiment. Participants were told that these adults were asked to imagine participating in an iterated trust game and were asked to indicate their choices between sending 20 points back and keeping all 40 points if they were entrusted 30 points by a stranger. Participants were also told that the experimenter recorded all these adults' choices and the computer would randomly select one adult's choices to respond to the participant's choice in the experiment. In reality, however, the only difference between the OTG and the ITG lies in the instruction. That is, all trustees' responses in both the OTG and the ITG were set up by a preprogrammed procedure (same across all participants), such that the decisions to reciprocate were made randomly across rounds, and the overall reinforcement rates for the trustor (i.e., the rates of receiving 20 points if the trustor makes the trusting choice) were approximately 50%.

### Stimuli and Procedure

**Figure 1:**
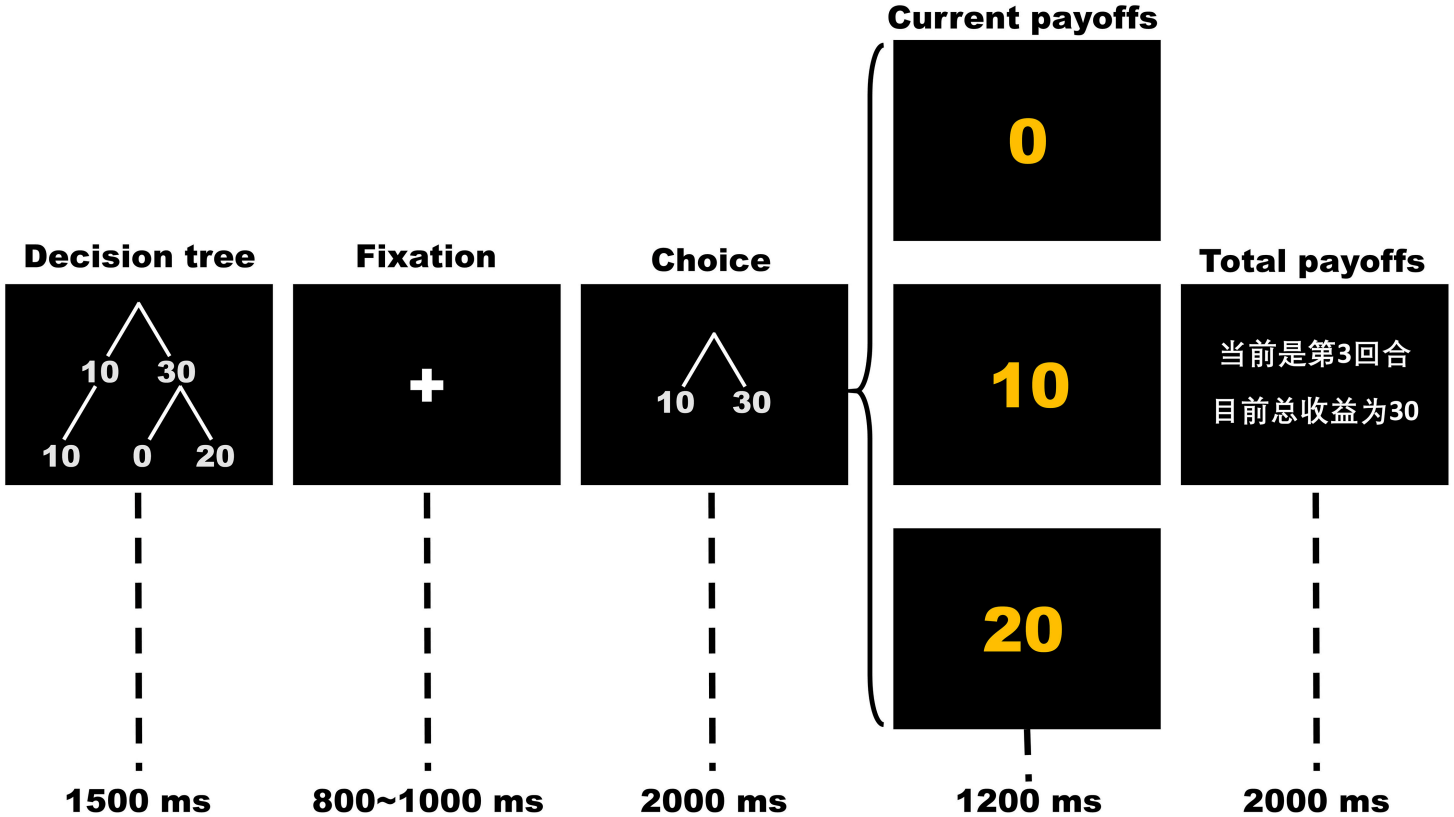
The time flow of each round in the trust game. The sample slide for total payoffs is written in Chinese. The text reads “The current trial is 3. Your total payoffs so far are 30”.

Participants completed 150 rounds of the OTG or the ITG while their brain potentials were recorded using EEG. In each round, as presented in Figure 1, the participant first sees a picture of a simplified decision tree showing possible outcomes for his/her single decision for 1,500 ms. After a variable 800~1,000 ms fixation cross, a picture indicating decision options is displayed in the center of the screen for 2,000 ms. During this time, the participant chooses either to keep (cued by the number “10”) or to send (cued by the number “30”) his/her initial endowment by using his/her index finger to press either the “1” or “3” key on the keyboard, respectively. The position of decision options (10 and 30) as well as their mappings to the keys were counterbalanced between participants. If the participant fails to respond within 2,000 ms, a warning message that indicating he/she responds too slowly would be displayed to the participant and the round will be restarted. Following a variable 800~1,200 ms inter-stimuli interval with a black screen, the outcome of the participant's current trial and his/her current total scores are displayed for 1,200 and 2,000 ms, respectively. The sample slide for total outcome-evaluation is written in Chinese. The text reads “The current trial is #3. Your total points so far are 30.” In the decision-making stage, the participant's choices to send 30 points (cued by “30” on the slide) indicate trust, whereas his/her choices to keep 10 points (cued by “10” on the slide) indicate distrust. In the outcome-evaluation stage, receiving 0 points (cued by “0”) is considered trust betrayal, receiving 10 points (cued by “10”) is considered a neutral condition and receiving 20 points (cued by “20”) is considered trust reciprocity.

Upon a participant's arrival, they were randomly divided into the OTG group or the ITG group, and then the experimenter described the rules of the trust game in detail. In particular, to make participants treat game points seriously, they were told that their participation compensation would be tied to the total number of game points earned in the game. However, regardless of their choices, participants earned a similar number of points over trials, and we actually paid them a flat rate for their participation (~8 dollars each person). Then, participants seated comfortably 1 m from a computer screen and were fitted with an electrode cap in an electromagnetically shielded room. Before the formal task, a practice block of 10 rounds was administered to familiarize the participants with the task procedures and to ensure that task instructions were understood.

## Data Acquisition and Processing

### Data Acquisition

We measured electrical brain activity from 64 channels using a modified 10–20 system electrode cap (Neuroscan Inc.). All EEG was recorded using a 0.05–100 Hz bandpass filter and continuously sampled at 1000 Hz with the right mastoid reference and a forehead ground. The vertical electrooculography (EOG) activity was recorded with electrodes placed above and below the left eye, and the horizontal EOG was recorded from two electrodes placed 1.5 cm lateral to the left and right external canthi. All electrode sites were cleaned with alcohol, and the impedance between electrodes and scalp was maintained below 5 kΩ.

### Data Preprocessing

**Figure 2:**
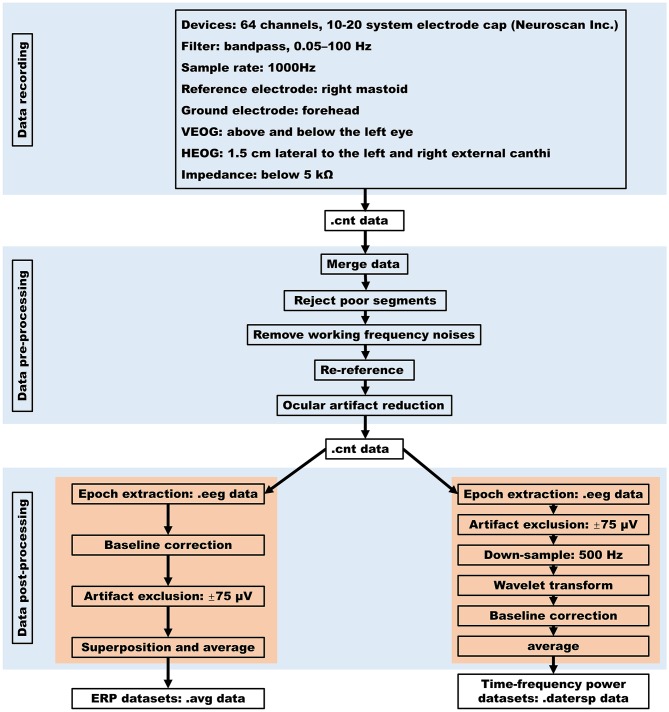
The flow diagram of data processing.

The flow diagram of data processing was presented in Figure 2. Using SCAN software (Neuroscan Inc.), the data preprocessing was performed in five steps. Firstly, the raw EEG data of each participant (i.e., cnt format) were merged with their mark data that contain their responses on each trial. Secondly, by visual observation to the .cnt data of each participant, the data segments with poor quality (e.g., obvious waveform drift) were rejected. Thirdly, in order to remove the working frequency (50 Hz) noises, the .cnt data of each participant were filtered with a 45~55 Hz bandstop filter with the zero phase shift model and a 24 db/oct attenuation slope. Fourthly, the .cnt data of each participant were re-referenced to the averaged bilateral mastoid. Finally, ocular artifacts in each participant's .cnt data were corrected with a regression-based eye-movement correction algorithm implemented in SCAN software (Semlitsch et al., 1986).

### Data Post-processing

To facilitate the reuse of our database, the .cnt data were further processed to get ERPs datasets and time-frequency power datasets.

### ERPs Datasets

Using SCAN software (Neuroscan Inc.), ERPs datasets were got in four steps separately for the decision-making stage and the outcome-evaluation stage. Specifically, for the decision-making stage (i.e., when the participant made a choice between keeping and sending the initial endowment), epochs were extracted from each participant's .cnt data from 200 ms before to 1,000 ms after each decision-making interface presentation at first. Then, the baseline correction was performed by subtracting the average value of epochs ranging from −200 to 0 ms (decision-making interface onset) from each epoch. After that, the epochs (trials) in which EEG voltages exceeded a threshold of ±75 μV during recording were excluded. Finally, effective trials of each participant were superposed and averaged for the two decision conditions (i.e., trust vs. distrust) and ERPs datasets (.avg format) of the decision-making stage were got. For the outcome-evaluation stage (i.e., when the participant sees the outcomes), epochs were extracted from each participant's .cnt data from 200 ms before to 1000 ms after each feedback presentation at first. Then, the baseline correction was performed by subtracting the average value of epochs ranging from −200 to 0 ms (outcome onset) from each epoch. After that, the epochs (trials) in which EEG voltages exceeded a threshold of ±75 μV during recording were excluded. Finally, effective trials of each participant were superposed and averaged for the two feedback conditions [i.e., trust reciprocity (gain) vs. trust betrayal (loss)] and ERPs datasets (.avg format) of the outcome-evaluation stage were got.

### Time-Frequency Power Datasets

Time-frequency power datasets were got in five steps separately for the decision-making stage and the outcome-evaluation stage. Firstly, single-trial epochs were extracted from each participant's continuous .cnt data to get .eeg data using SCAN software separately for the decision-making and the outcome-evaluation stages. For the decision-making stage (i.e., when the participant made a choice between keeping and sending the initial endowment), single-trial epochs were extracted from 1,000 ms prestimulus to 2,000 ms poststimulus after each decision-making interface presented. For the outcome-evaluation stage (i.e., when the participant sees the outcomes), single-trial epochs were extracted from 1,000 ms before to 2,000 ms after each feedback presentation. Secondly, using SCAN software (Neuroscan Inc.), the average value of epochs ranging from −200 to 0 ms was subtracted from each epoch and artifact exclusion on .eeg data was performed so that the epochs (trials) in which EEG voltages exceeded a threshold of ±75 μV during recording were excluded. Thirdly, the .eeg data were downsampled to 500 Hz, and a Morlet-based wavelet transform procedure implemented in EEGLAB (Version 14_1_1b) was employed. By which, the continuous estimate of time-frequency power in a given frequency band (3–35 Hz) as a function of time between −1,000 and 2,000 ms was obtained (Delorme and Makeig, 2004). Fourthly, time-frequency power was normalized with respect to a 400 to 200 ms prestimulus baseline and converted to decibels [10 × log (μV^2^)]. Finally, the time-frequency power among multiple trials of the same condition (trust or distrust condition for the decision-making stage; gain or loss condition for the outcome-evaluation stage) were averaged (Makeig et al., 2004), and the time-frequency power datasets (.datersp format) were obtained.

### Data Validation

To validate the quality of the current database, we further examined the grand ERPs waveforms of ERPs datasets and spectral power maps of time-frequency power datasets, respectively. For ERPs datasets, the grand average ERPs waveforms and the corresponding scalp topographies of 16 representative electrodes in the decision-making phase and outcome evaluation phase were gained and shown in Figures S1–S4. For each ERP, 0 ms indicates the onset of the decision-making/outcome feedback stimulus, and activity in the −200 to 0 ms time window prior to the decision-making/outcome feedback stimulus served as the baseline. For time-frequency power datasets, the spectral power maps of five representative electrodes were gained and shown in Figures S5, S6 in the decision-making and outcome feedback phases, respectively. The results indicated that the stable ERPs waveforms and spectral power maps can be gained through the current datasets, which thus assured the data quality of the current database.

### Usage Notes

As presented in Table S1, the current database contains three zip files and a pdf file. The raw EEG datasets (.cnt format) without any preprocessing or conversion were saved in the zip file named Raw EEG data. The ERPs datasets (.avg format) of the decision-making stage and the outcome-evaluation stage were saved in the zip file named Average waveform data. The time-frequency power datasets (.datersp format) of the decision-making stage and the outcome-evaluation stage were saved in the zip file named Spectral power data. The demographic and behavioral data of participants were saved in the pdf file named demographic and behavioral data. All files were named by the participants' code and file modality. As an example, the file names of participant 01 were listed in the last column of Table S1. All these datasets can be freely downloaded from the open-access repository (Dryad Digital Repository, https://datadryad.org/stash/share/I4_9eQgXJL0sjukj8I9ruT2TToTZ90RuZvSmJO5LnyY). Previous studies have proposed that the one-shot trust and iterated trust simulated the generalized trust between strangers and specific trust between acquaintances, respectively (Rousseau et al., 1998; Buskens et al., 2016). In this sense, the current database may be used to examine the potential neural-dynamical differences between these two basic trust modes. Given that a priori power analysis suggested that 17 participants per group would allow detection of a large effect (Cohen's d = 1.00) with.80 power and.05 Type I error rate (Faul et al., 2007; Mayr et al., 2007), the current number of participants is enough for above potential usages. We encourage potential users from other laboratories or organizations to use the current database under the requirement of citing the present data report. At the same time, we also hope that all users of the database will acknowledge the original authors by citing this publication.

## Data Availability Statement

All these datasets can be freely downloaded from the open-access repository Dryad Digital Repository, https://datadryad.org/stash/share/I4_9eQgXJL0sjukj8I9ruT2TToTZ90RuZvSmJO5LnyY.

## Ethics Statement

The studies involving human participants were reviewed and approved by Ethics Committee of Psychological and Cognitive Sciences of Fuzhou University. The patients/participants provided their written informed consent to participate in this study.

## Author Contributions

YW, JL, and LZ contributed conception and design of the study. CF collected the data and drafted the manuscript under the supervision of XYao and XYan. YW and JL provided critical revisions. All authors contributed to manuscript revision, read, and approved the submitted version.

### Conflict of Interest

The authors declare that the research was conducted in the absence of any commercial or financial relationships that could be construed as a potential conflict of interest.
