# Supplementary material for: Trust Game Database: Behavioral and EEG Data From Two Trust Games
Source: Front Psychol. 2019 Dec 12;10:2656. doi: 10.3389/fpsyg.2019.02656 (PMC6920181; doi:10.3389/fpsyg.2019.02656)
Supplement: Supplementary file 1 [file Data_Sheet_1.PDF]

Supplementary materials

Table S1. Specification for the Trust Game Database.

Name of zip file	Folder name	Condition	Number of files	Filename of participant 01
Spectral power data	One-shot-trust-ERSP	trust	20	sub01_one_trust.datersp
	One-shot-distrust-ERSP	distrust	20	sub01_one_dis.datersp
	One-shot-gain-ERSP	gain	20	sub01_one_gain.datersp
	One-shot-loss-ERSP	loss	20	sub01_one_loss.datersp
	Iterated-trust-ERSP	trust	20	sub01_re_trust.datersp
	Iterated-distrust-ERSP	distrust	20	sub01_re_dis.datersp
	Iterated-gain-ERSP	gain	20	sub01_re_gain.datersp
	Iterated-loss-ERSP	loss	20	sub01_re_loss.datersp
Average waveform data	One-shot-trust-distrust -difference (decision-making stage)	trust	20	sub01-trust-68.avg
		distrust	20	sub01-distrust-68.avg
		distrust-trust	20	sub01-dis-trust-68.avg
	One-shot-gain-loss -difference (outcome-evaluation stage)	gain	20	sub01_gain.avg
		loss	20	sub01_loss.avg
		Loss-gain	20	sub01_loss-gain.avg
	Iterated-trust-distrust -difference (decision-making stage)	trust	20	sub01-trust-39.avg
		distrust	20	sub01-distrust-40.avg
		Distrust-trust	20	sub01-dis-trust-40.avg
	Iterated -gain-loss-difference (outcome-evaluation stage)	gain	20	sub01_gain.avg
		loss	20	sub01_loss.avg
		Loss-gain	20	sub01_dFRN.avg
Raw EEG data	One-shot-cnt	One-shot	20	One_shot_raw_eegdata_sub01.rar
	Iterated-cnt	Iterated	20	Iterated_raw_eegdata_sub01.rar
Demographic and behavioral data		demographic and behavioral data.pdf		

.

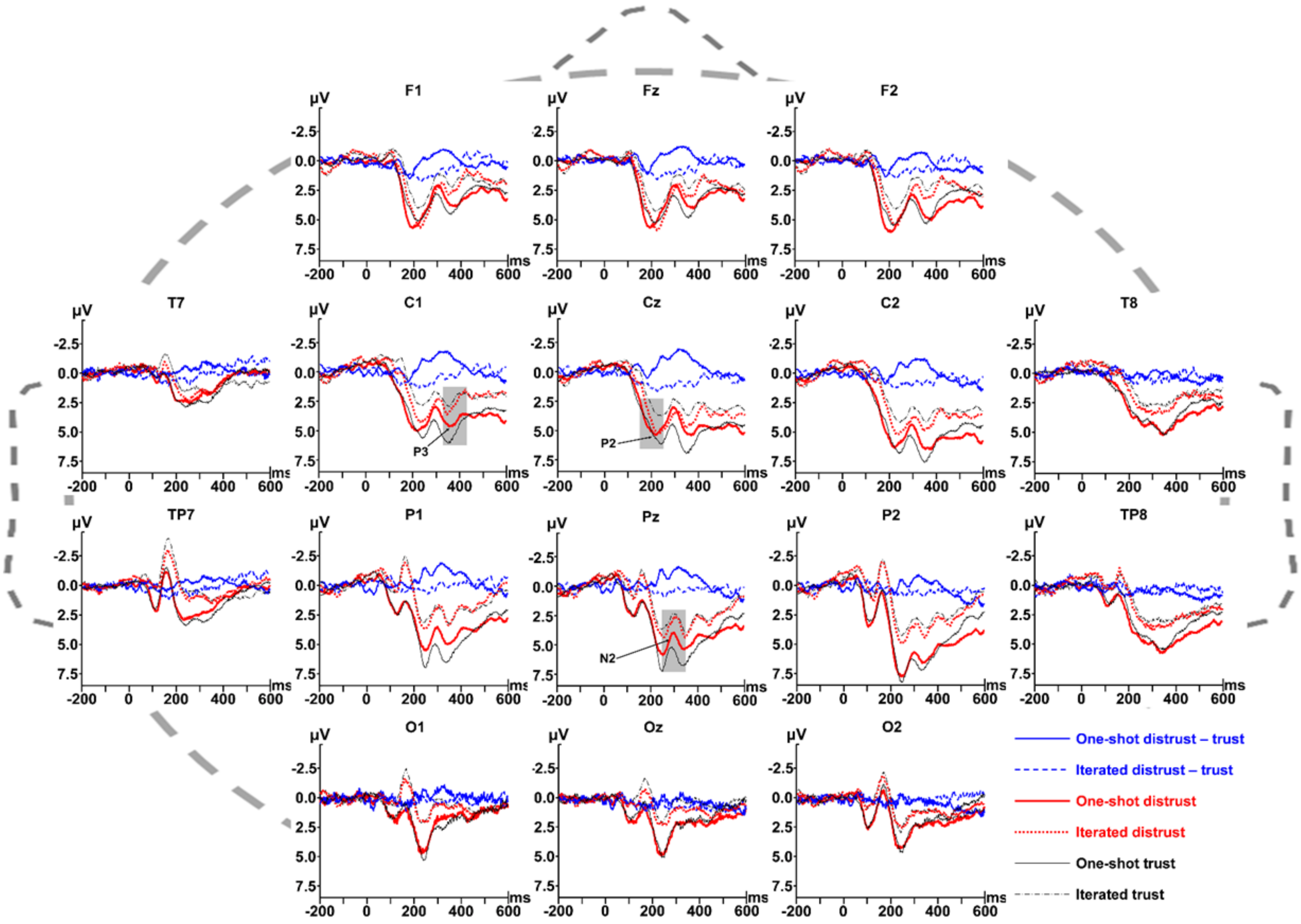

Figure S1. Grand average ERP waveforms of one-shot trust game and iterated trust game in the decision-making phases. The x-axis represents the timecourse in millisecond (ms), and the y-axis represents the amplitude of waveforms in microvolts (μV).

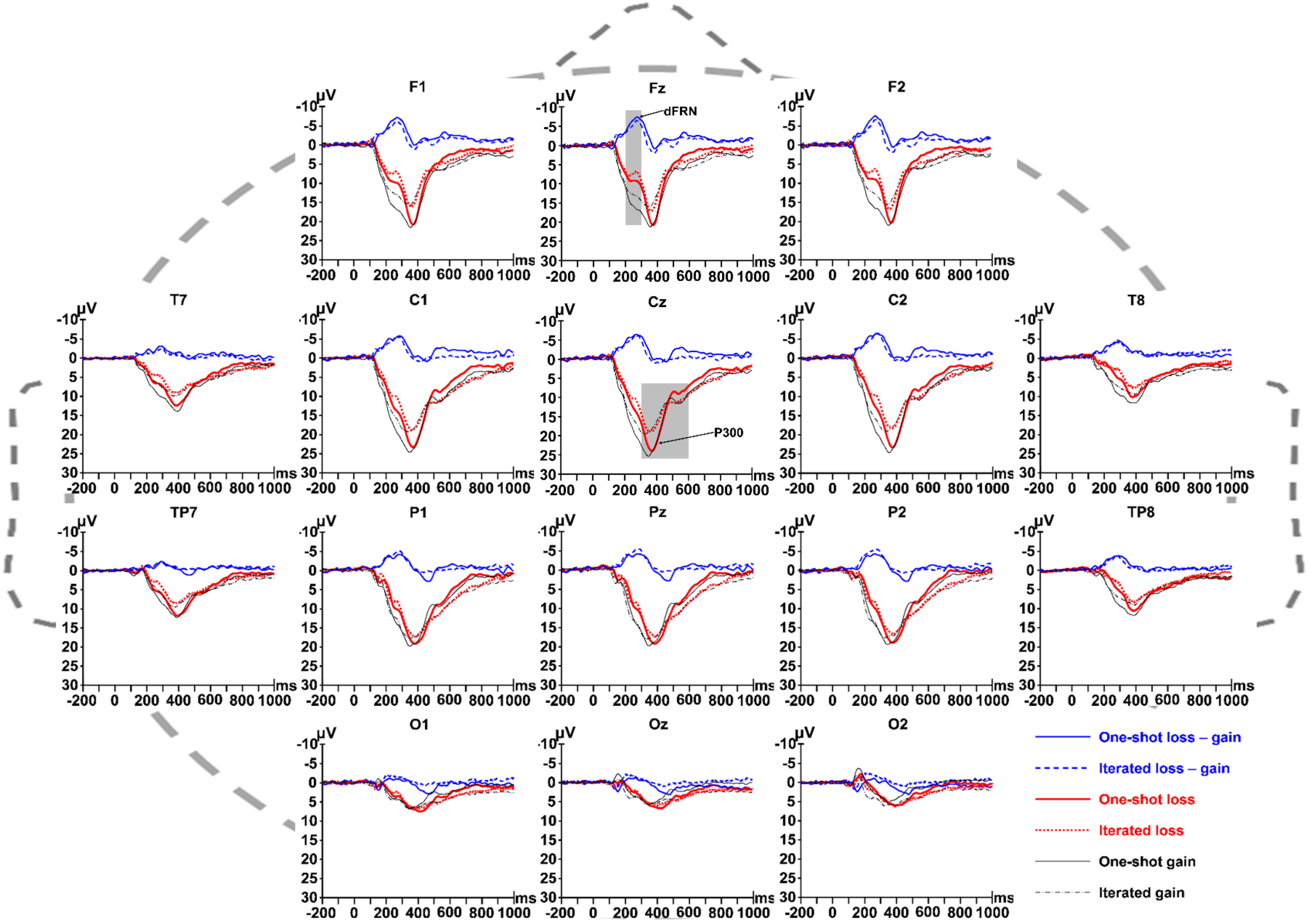

Figure S2. Grand average ERP waveforms of one-shot trust game and iterated trust game in the outcome feedback phases. The x-axis represents the timecourse in millisecond (ms), and the y-axis represents the amplitude of waveforms in microvolts (μV).

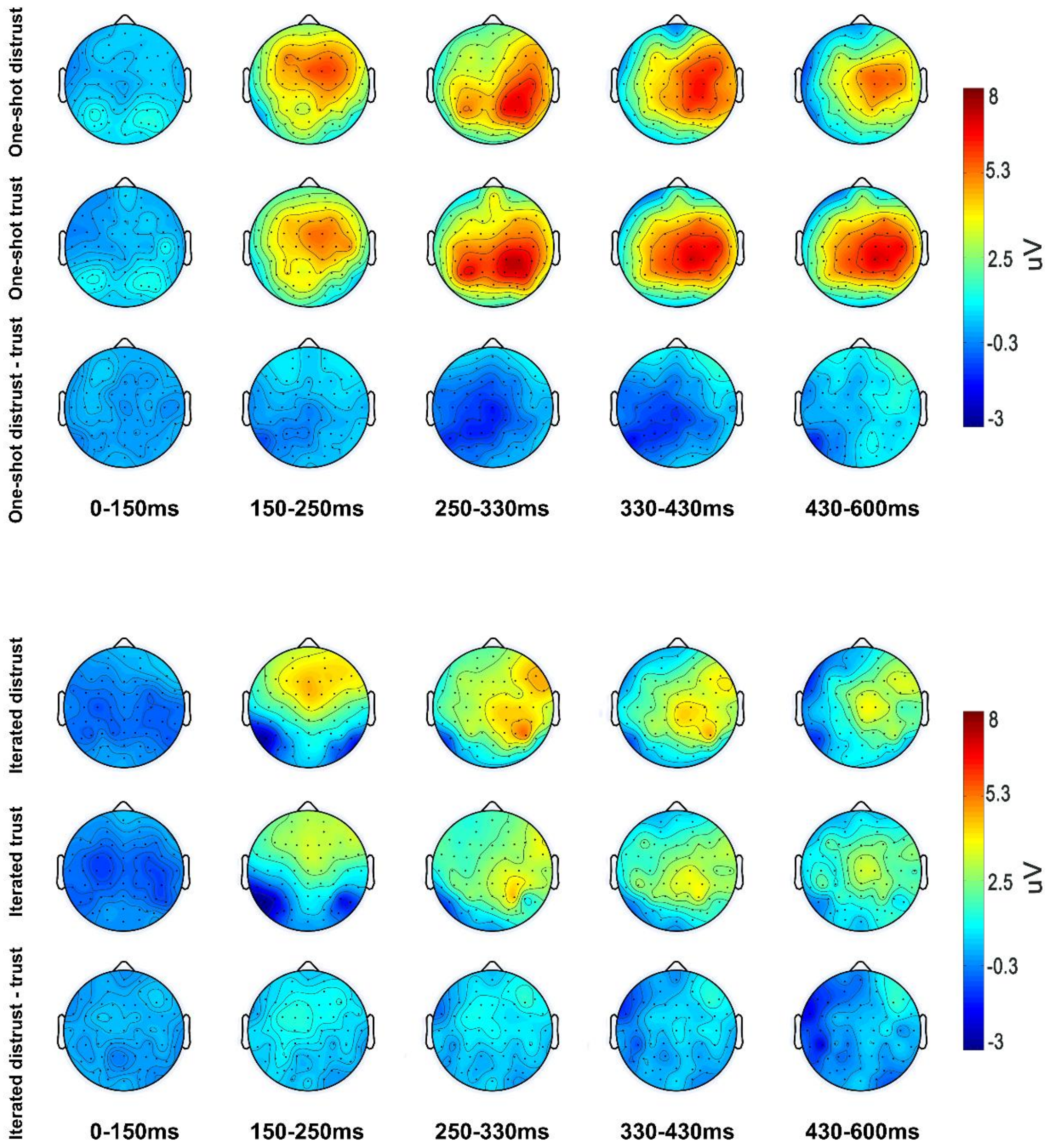

Figure S3. Spatial distribution of amplitudes across the scalp of one-shot trust game (top) and iterated trust game (bottom) in decision-making phases. The x-axis represents time windows in millisecond (ms), and the color bar (unit: μV) represents the average amplitude of waveforms in corresponding time windows.

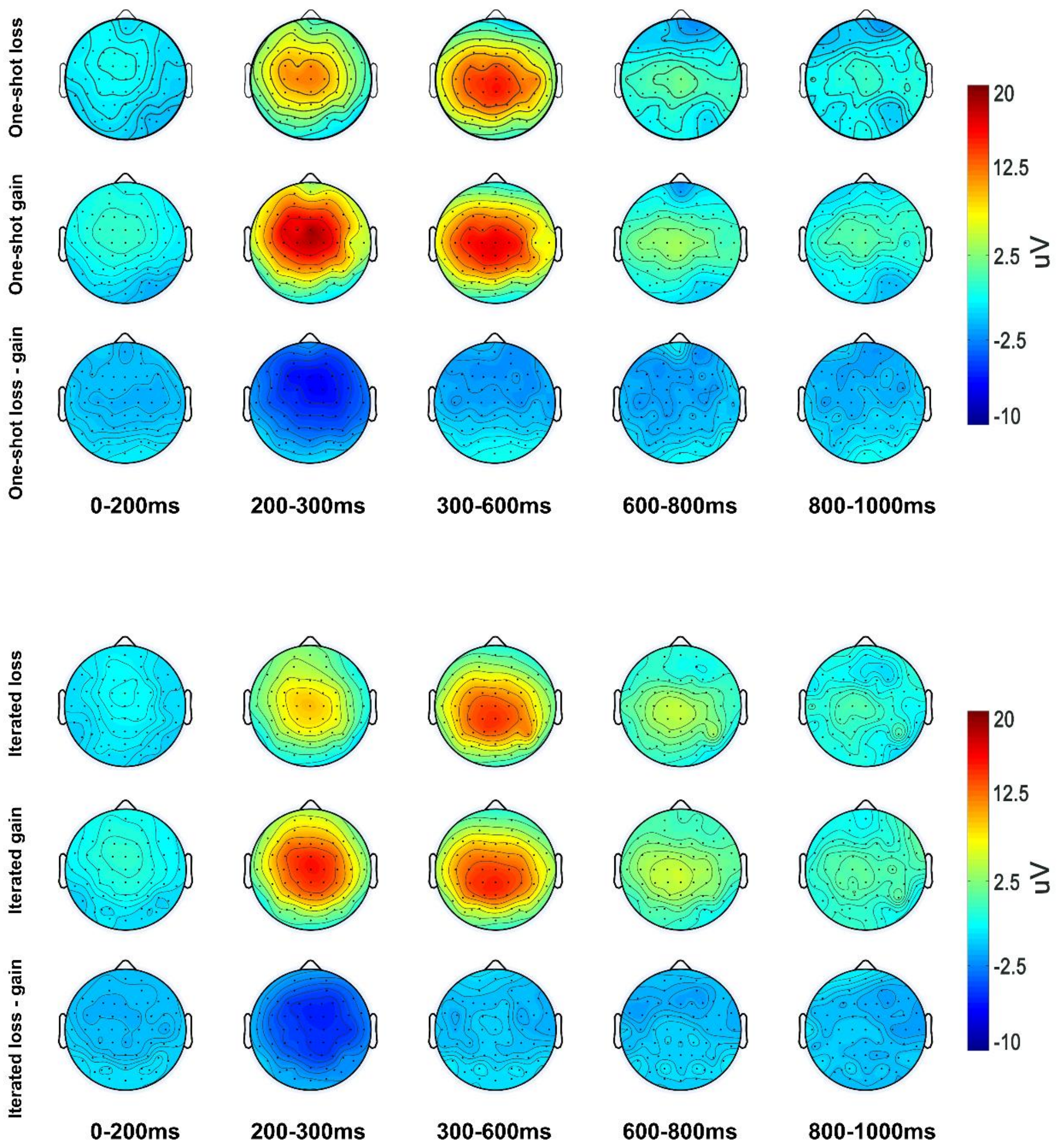

Figure S4. Spatial distribution of amplitudes across the scalp of one-shot trust game (top) and iterated trust game (bottom) in outcome feedback phases. The x-axis represents time windows in millisecond (ms), and the color bar (unit: μV) represents the average amplitude of waveforms in corresponding time windows.

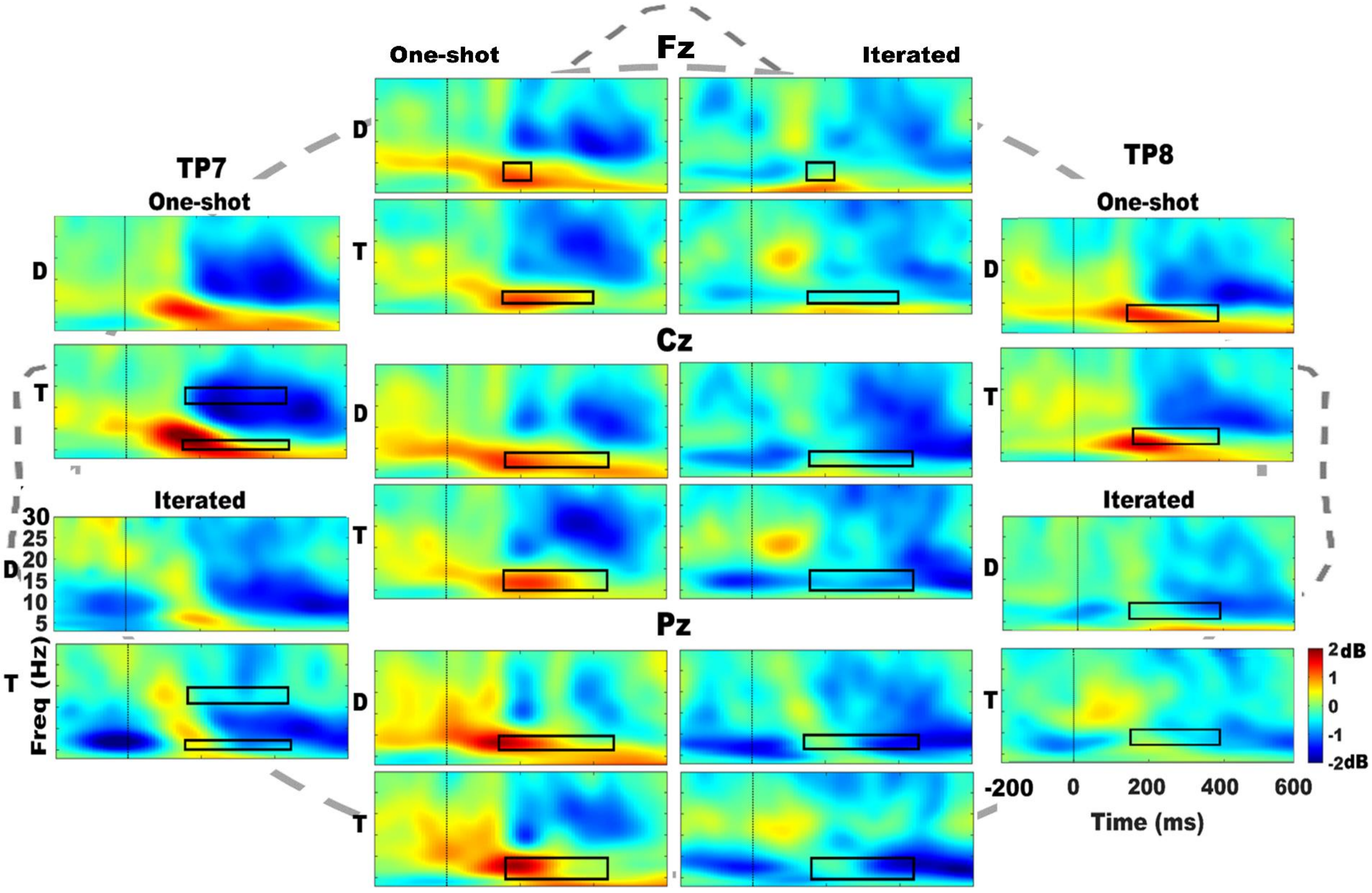

Figure S5. Spectral power (unit: dB) of the one-shot trust game and iterated trust game in decision-making phases. The x-axis represents the time course in millisecond (ms), and the y-axis represents the frequency in hertz (Hz). Vertical black dashed lines indicate the onset. D = distrust; T = trust.

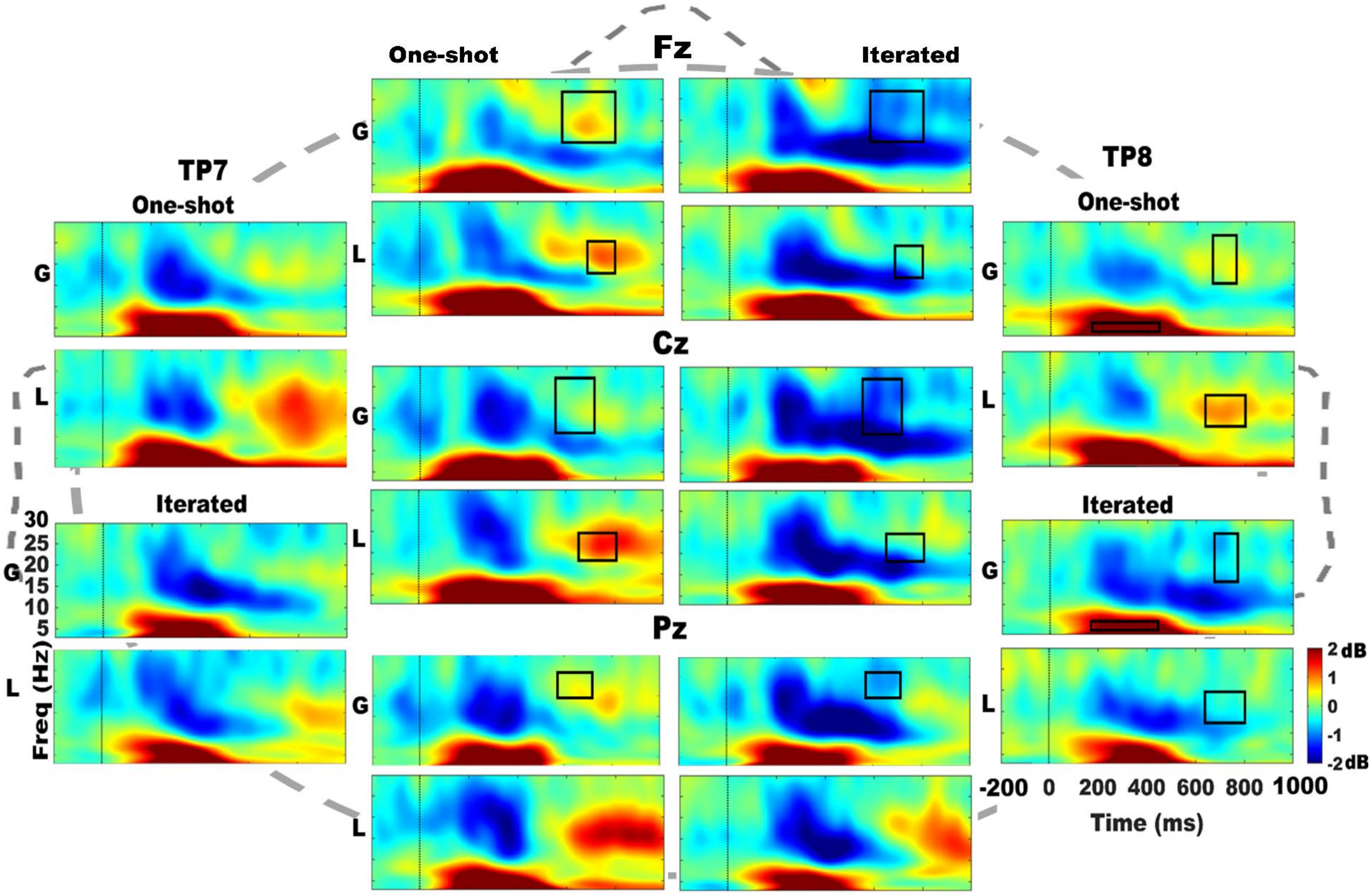

Figure S6. Spectral power (unit: dB) of the one-shot trust game and iterated trust game in outcome feedback phases. The x-axis represents the time course in millisecond (ms), and the y-axis represents the frequency in hertz (Hz). Vertical black dashed lines indicate the onset. G = gain; L = loss.
